# Early microbial colonization study of daily-use plastics exposed to river water

**DOI:** 10.1007/s11274-026-04907-z

**Published:** 2026-04-18

**Authors:** Dinesh Parida, Swagata Lakshmi Dhali, Kiran Bala, Regina Nogueira

**Affiliations:** 1 Mehta Family School of Biosciences and Biomedical Engineering, Indian Institute of Technology Indore, Simrol, Madhya Pradesh 453552 India; 2 Institute of Sanitary Engineering and Waste Management, Leibniz University Hannover, Welfengarten 1, Hannover, 30167 Germany; 3 Mehta Family School of Sustainability, Indian Institute of Technology Indore, Simrol, Madhya Pradesh 453552 India

**Keywords:** Plastisphere, River microcosm, Structural metagenomics, Synthetic polymers, Microbial colonization

## Abstract

In rivers, microorganisms colonize plastic surfaces, initiating processes that can lead to their microbial decomposition. Our study investigates the bacterial community composition and diversity on the surfaces of plastics used daily, such as polyethylene terephthalate (PET) and low-density polyethylene (LDPE), which were exposed to river water from the Aller and Fusche rivers. Glass was used for comparison purposes. 16s rRNA sequencing revealed that the type of surface and the native microbial community in the river water, including the water quality, significantly influenced biofilm community assembly. River water samples, especially from the Fusche site, supported the highest microbial richness, while plastic exhibited moderate diversity, and glass beads hosted the lowest richness and diversity. Proteobacteria and Bacteroidetes dominated across all samples, with notable enrichment of functionally relevant families such as Rhodobacteraceae and Comamonadaceae. Ecologically relevant genera such as *Flavobacterium*, *Hydrogenophaga*, *Rhodoferax*, *Sediminibacterium*, and *Rhodobacter* dominated across samples. Alpha diversity reflected the richness of taxa within each sample, while beta diversity revealed distinct clustering based on both plastic type and site, indicating the influence of ecological pressure and niche partitioning. These findings highlight the capacity of plastic surfaces to harbour diverse and specialised bacterial assemblages, with implications for biogeochemical cycling, pollutant interactions, and potential microbial degradation pathways. This work contributes to deciphering the ecological roles of biofilms in freshwater plastisphere micro-environments and underscores the importance of material-specific microbial dynamics in assessing environmental risks.

**Supplementary Information:**

The online version contains supplementary material available at 10.1007/s11274-026-04907-z.

## Introduction

Among the most prevalent polymers detected in aquatic environments, Polyethylene terephthalate (PET) and low-density polyethylene (LDPE), both are extensively used in packaging, textiles, and consumer goods (Bellasi et al. 2020). Their high chemical stability, hydrophobicity, and resistance to biological degradation make them particularly persistent in the environment (Chen et al. 2012; Olam 2023). Once these plastic materials enter freshwater systems, they do not remain inert; instead, they rapidly become colonised by a wide range of microorganisms, forming biofilms that mark the onset of a dynamic interaction between synthetic materials and the microbial community (Zettler et al. 2013; Harrison et al. 2014). Furthermore, it is still unclear whether the microbial communities exhibit material preference, i.e., whether different polymer types recruit distinct microbial assemblages or similar.

The colonised surface of plastics, often termed the plastisphere, represents a microhabitat distinct from the surrounding water column and natural surfaces (Amaral-Zettler et al. 2020). It comprises diverse microbial communities, including bacteria, fungi, algae, and protozoa. These communities can be shaped by multiple factors, including the physicochemical properties of the plastic (e.g., surface roughness, hydrophobicity, and chemical composition) and the duration of exposure (Zettler et al. 2013). The development of biofilms on plastic surfaces has ecological consequences, influencing microbial dispersal (Debroy et al. 2022), nutrient cycling, and potentially altering the fate and transport of plastics in aquatic systems (Mincer et al. 2016). Moreover, the plastisphere may serve as a reservoir for antibiotic resistance genes and pathogenic taxa, posing additional environmental and public health concerns (A and G 2022).

Recent research has begun to explore not just the composition of plastisphere communities, but also their functional roles, particularly their potential in mediating plastic degradation. Microbes secrete extracellular enzymes that deteriorate MPs into oligomers, dimers, and monomers. This process modifies the density, crystallinity, and surface morphology of the plastics, which eventually affects the MP distribution patterns in aquatic ecosystems (Zhang et al. 2024). Certain microorganisms, especially bacteria and fungi, have been reported to produce extracellular enzymes capable of acting on polymeric chains, resulting in surface erosion, functional group modification, or even partial mineralization of plastic substrates under specific conditions. A study conducted by Žorža et al., examined biofilm formation on PET plastics incubated in landfill leachate and enriched in complex media, revealing shifts in microbial communities from *Nitrosomonas* dominance in leachate to high *Pseudomonas* abundance after enrichment. Fluorescent in situ hybridisation (FISH) microscopy confirmed *Pseudomonas* distribution in biofilms. Enrichment conditions significantly influenced microbial composition and enzymatic activity (Žorža et al. 2025). In another study, researchers incubated smooth and rough high-density polyethylene (HDPE) along with wood in freshwater streams to study biofilm development over 56 days. Plastic surfaces supported lower species richness compared to wood, though rough HDPE had higher evenness. The stream site had a stronger influence on microbial composition than plastic texture, and biofilm differences between plastic and wood increased over time (Lopez Avila et al. 2025). However, the extent and ecological relevance of such a plastisphere in natural freshwater systems remains poorly understood.

PET, being a thermoplastic polyester, has a more crystalline structure and different surface energy than LDPE, which is a highly flexible, branched polyolefin. These differences could affect biofilm formation and microbial adhesion, possibly selecting for substrate-specific colonizers. In laboratory and marine studies, some degree of substrate specificity has been observed, but data from freshwater ecosystems, especially riverine systems subjected to different anthropogenic and hydrological influences, are still limited.

In this study, we focus on the early-stage plastisphere development on two common polymers: PET and LDPE in river water from the Aller and Fusche rivers. By comparing microbial communities developed on the two polymers, we aim to answer the following key questions:

i. Do microbial communities on plastic surfaces differ significantly from one another depending on the polymer chemical composition?
ii. What types of bacterial communities are potentially associated with early-stage plastic colonization?
iii. Do the two rivers have similar microbial communities at their sources? If so, how do community dynamics differ with plastic inclusion?

The outcomes of this study will contribute to a deeper understanding of the ecological role of microbial communities in the freshwater plastisphere. It may also help identify microbial indicators of plastic decomposition.

## Materials and methods

### Sampling location and collection

Surface water samples were collected from the Aller (52°35’16.2"N, 10°08’26.5"E) and Fusche (52°34’00.4"N, 10°06’29.6"E) rivers from the town of Celle in Hannover, Germany. The Aller River flows through southeastern Germany, eventually joining the Weser River near Verden. The Aller is commonly used for recreational activities like boating, fishing, canoeing, and water skiing. One of its main tributaries is the Fusche river, which gets its water from the Harz foreland, not from the Harz mountains directly. Both these rivers have different water chemistry and niche microbial seed pools (Band 2003; Gew and Faasch 2004). A detailed description of the sampling locations is provided in Fig. S1.

### Plastic samples

Two types of MPs, LDPE films and PET sheets, were cut to the same dimensions (5 mm x 5 mm). The LDPE MP films were procured from single-use plastic bags from a grocery store in Germany, and the PET MPs were obtained by cutting a German commercial soft drink bottle. The detailed chemical characterization of the two MPs used in the experiment was done by Attenuated total reflectance Fourier-transform infrared spectroscopy (ATR-FTIR).

### TTC viability assay using River water

The TTC salt viability assay was performed according to the methodology given in Khandare et al. (Khandare et al. 2021). To screen the viability of microbes in the river water, 2,3,5-triphenyl tetrazolium chloride (TTC) salt was used to indicate bacterial viability in a carbon-free medium. Instead of a medium, river water from Aller and Fusche was used. The LDPE and PET MPs fragments were weighed and sterilized by first incubating in 70% ethanol solution, drying them, and finally UV sterilizing for 20 min. In 20 ml culture tubes, 10 mL of river water, 20 µL of 1% filter-sterilized (using filter paper of pore size 0.22 μm) TTC salt solution were added. The tubes were kept in a shaking condition at 220 rpm for 5 days. After 5 days, the colour change was observed visually. Based on this, a 21-days incubation experiment was carried out, and biofilm formation was subsequently evaluated over the same 21- days period.

### Fluorescence microscopy for live/ dead staining

Micrographs were acquired using a fluorescence microscope (Zeiss Apotome 2, Blue Edition). For the live/ dead cells observation, one MP particle was taken out of each flask each week for three weeks and stained according to the manufacturer’s instructions using the LIVE/DEAD BacLightTM Bacterial Viability Kit (Molecular Probes by Life Technologies, USA). To avoid non-specific dye binding, the excess stain was removed after incubation, and the MPs containing biofilms were rinsed three times with filter-sterilized DI water. The excitation/emission maxima for SYTO 9 dye are 480/500 nm, while those of propidium iodide are 490/635 nm; hence, appropriate channels were used for observation.

### Experimental set-up

The LDPE and PET MPs were prepared for the experiment by sterilizing them in 70% ethanol solution for 1 h. These were subsequently rinsed with sterile deionized water and inoculated in flasks using sterile pincers. As a control substrate for the experiment, cover slip glass was cut into the same dimensions (5 mm x 5 mm) and inoculated into separate flasks (negative controls). To standardize the surface area accessible for colonization, the total surface area of each material type was kept constant (~ 100 mm^2^) across each flask (Ogonowski et al. 2018). 250 mL (Schott Duran) flasks were filled with 150 mL of river water collected from the Aller and Fusche Rivers. To obtain a representative sample of the bacterial community, 150 mL of the river water from both rivers was vacuum-filtered on a 0.22 μm Millipore filter (Millipore, USA); the filter was used for DNA extraction. 4 pieces of each substrate type (PET, LDPE, and glass) were incubated in 150 mL of each river water so that ~ 100 mm^2^ surface area was available in each flask for colonization. The flasks were incubated at room temperature (~ 25 °C) on the windowsill of the lab. The average UV index was 4. To promote aerobic conditions, the flasks were covered with loose plugs and stirred manually 2–3 times each week for 1–2 min. Each week for three consecutive weeks, three flasks containing each river type were sacrificed for biofilm quantification and visualization.

### DNA extraction, purification, library preparation, and Illumina MiSeq sequencing

Bacterial DNA was extracted using the NucleoMag^®^ DNA/RNA Water kit following the manufacturer’s instructions. The obtained DNA was purified using the Zymo Research Genomic DNA Clean & ConcentratorTM-10.

Next-generation sequencing library preparations and Illumina MiSeq sequencing were conducted at GENEWIZ, Inc. (Suzhou, China). DNA samples were quantified using a Qubit 2.0 Fluorometer (Invitrogen, Carlsbad, CA, USA). 30–50 ng DNA was used to generate amplicons using a MetaVx™ Library Preparation kit (GENEWIZ, Inc., South Plainfield, NJ, USA). V3, V4, and V5 hypervariable regions of prokaryotic 16S rDNA were selected for generating amplicons and following taxonomy analysis. GENEWIZ designed a panel of proprietary primers aimed at relatively conserved regions bordering the V3, V4, and V5 hypervariable regions of bacteria and Archaea16S rDNA. The v3 and v4 regions were amplified using forward primers containing the sequence “CCTACGGRRBGCASCAGKVRVGAAT” and reverse primers containing the sequence “GGACTACNVGGGTWTCTAATCC”. The v4 and v5 regions were amplified using forward primers containing the sequence” GTGYCAGCMGCCGCGGTAA” and reverse primers containing the sequence “CTTGTGCGGKCCCCCGYCAATTC”. 1st round PCR products were used as templates for the 2nd round amplicon enrichment PCR. At the same time, indexed adapters were added to the ends of the 16 S rDNA amplicons to generate indexed libraries ready for downstream NGS sequencing on Illumina MiSeq.

DNA libraries were validated by Agilent 2100 Bioanalyzer (Agilent Technologies, Palo Alto, CA, USA) and quantified by Qubit 2.0 Fluorometer. DNA libraries were multiplexed and loaded on an Illumina MiSeq instrument according to the manufacturer’s instructions (Illumina, San Diego, CA, USA). Sequencing was performed using a 2 × 300/250 paired-end (PE) configuration; image analysis and base calling were conducted by the MiSeq Control Software (MCS) embedded in the MiSeq instrument.

**Fig. 1:**
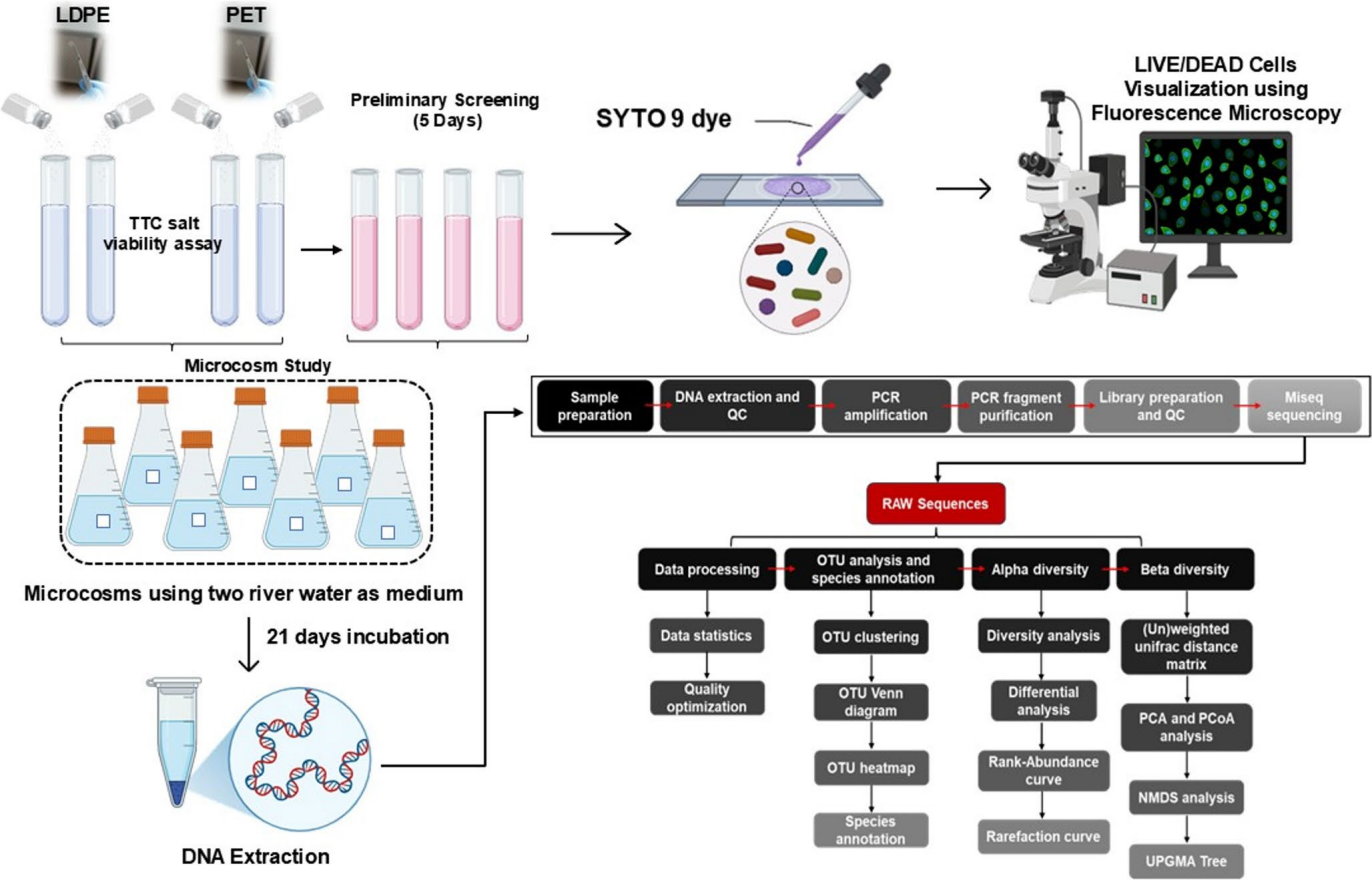
Overview of the methodology involving steps from preliminary plastic treatment to sequencing and data analysis

A detailed overview of the methodology is given in Fig. 1.

### Data analysis

The QIIME data analysis package was used for 16 S rRNA data analysis (Lawley and Tannock 2017). The forward and reverse reads were joined and assigned to samples based on barcode and truncated by cutting off the barcode and primer sequence. Quality filtering on joined sequences was performed, and sequences that did not fulfil the following criteria were discarded: sequence length < 200 bp, no ambiguous bases, and mean quality score ≥ 20. Then the sequences were compared with the reference database (RDP Gold database) using the UCHIME algorithm to detect chimeric sequences, and then the chimeric sequences were removed. The effective sequences were used in the final analysis. Sequences were grouped into operational taxonomic units (OTUs) using the clustering program VSEARCH (1.9.6) against the Silva 119 database pre-clustered at 97% sequence identity. The Ribosomal Database Program (RDP) classifier was used to assign taxonomic categories to all OTUs at a confidence threshold of 0.8. The RDP classifier uses the Silva 119 database, which has taxonomic categories predicted to the species level.

### Quality control/ quality assurance

Staining dyes were prepared in a glass bottle to avoid cross-contamination from the plastic bottles. Lint-free tissue papers were used during the experimentation. During the experiments, we have used cotton lab coats as protective gear. DNA isolation and staining procedures were performed inside the laminar air-flow to avoid contamination.

## Result and discussion

### Preliminary insights from the TTC viability assay and fluorescence microscopy

**Fig. 2:**
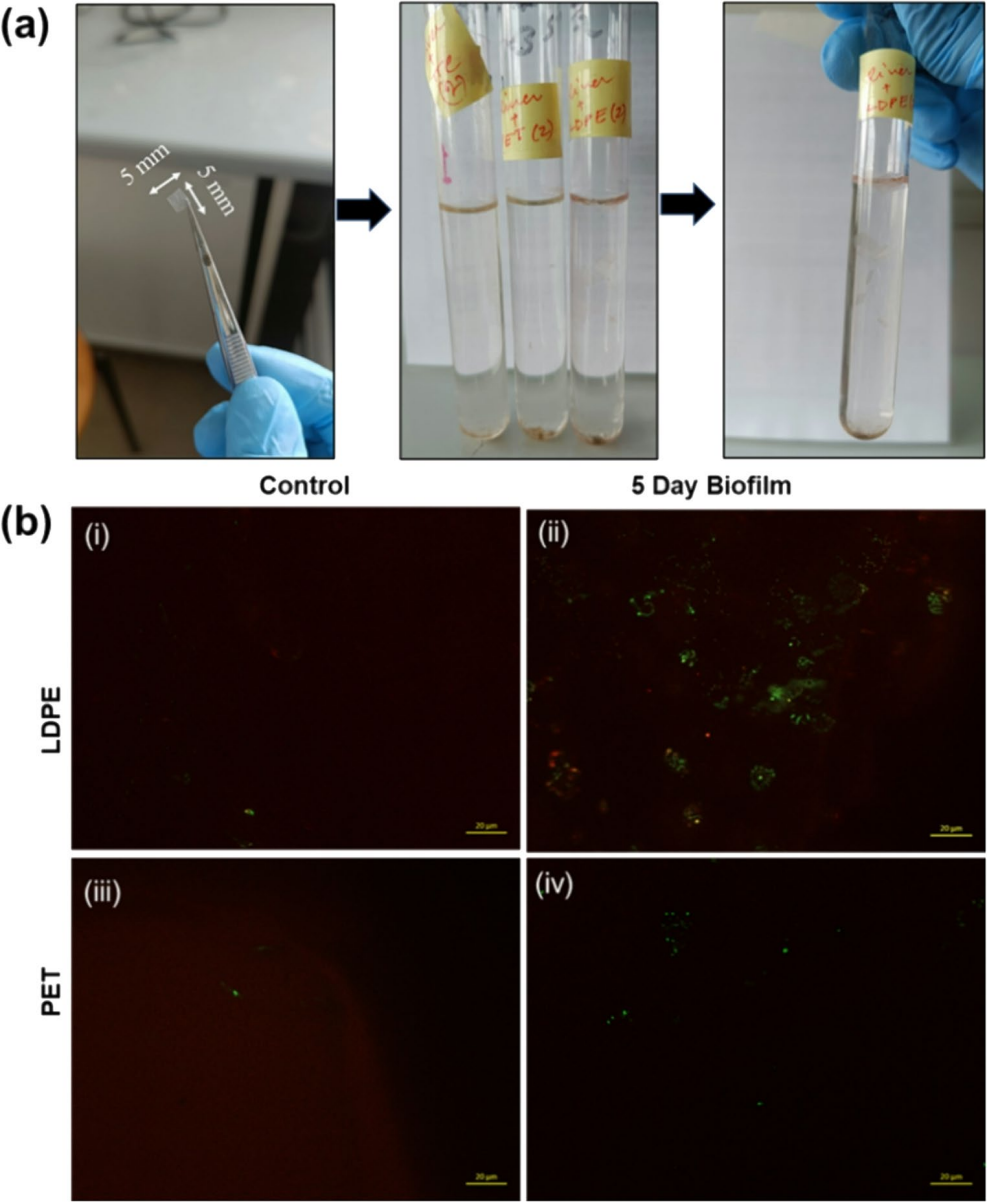
**a** TTC salt viability assay using LDPE and PET MPs as a substrate in Aller river water, demonstrating microbial activity. The pink colouration reflects active microbial colonization and viability on the plastic surfaces; **b** Fluorescence micrographs show biofilm formation on LDPE and PET MPs surfaces incubated in Aller River water supplemented with TTC salt as a viability indicator; Fluorescence micrographs depicting the live and dead cells on the LDPE and PET surfaces after five-day incubation in control (i, iii) and in river water medium (ii, iv) (The similar test was performed using Fusche river water too, here in the figure only Aller biofilm is represented)

After incubating the LDPE and PET sheets in the two-river water for five days, a slight colour change was observed in both tubes, from transparent to pink. The LDPE films appeared to be partially sinking through the tube after five days, but they tend to float in their initial form, confirming the colonization- induced increase in density. As PET has a higher density, sinking was observed in the initial phase of the experiment along with the colour change in the medium. Due to the colour shift, a fluorescent kit was used for live/dead cell staining. As we can see clearly in Fig. 2, the results indicated that there were more green dots representing living cells and the red spots signifying dead cells. In the LDPE samples, more green spots can be observed in comparison to the PET biofilms. After this initial screening, it was confirmed that the microbial community present in both the river water can colonize the plastic substrate. Similar findings were reported by Khandare et al. (2025) and Taghavi et al. (2021) in their studies conducted on Polyvinyl chloride (PVC), Polyethylene terephthalate (PET), Polystyrene (PS), and Polyethylene (PE) (Taghavi et al. 2021; Khandare et al. 2025).

Subsequent fluorescence microscopy analysis of the biofilms from the experimental flasks enabled progressive visualization of live and dead cells within the developing biofilms. Over a time period, a gradual increase in dead cells was observed, and by the 21^st^ day, the biofilms were dominated by non-viable cells (represented by red dots in Fig. S2). The micrographs indicate that the density of the viable cells reached a plateau phase, where no further increase in viable cells or expansion of the biofilm structure was apparent. This stabilization in cells suggests that active biofilm development had ceased by this stage. Therefore, to confirm whether biofilm formation was still progressing or had reached a stationary state, the observations were extended up to the 21st day of incubation. The lack of noticeable increase in viable cells or structural biofilm growth during this period supports the conclusion that the biofilm had entered a mature or decline phase characterized by reduced cellular viability. Accordingly, the 21^st^ day was selected as the final time point for observation, after which the samples were processed for DNA extraction. As no external Carbon source was supplied, the initial microbial community likely relied primarily on the plastic surface and residual nutrients, leading to substrate exhaustion over time (De Plano et al. 2024). This depletion is consistent with the increased prevalence of red fluorescence, indicating a higher proportion of dead cells at later stages of incubation.

### Structure of bacterial communities

#### Broad perspective

**Fig. 3:**
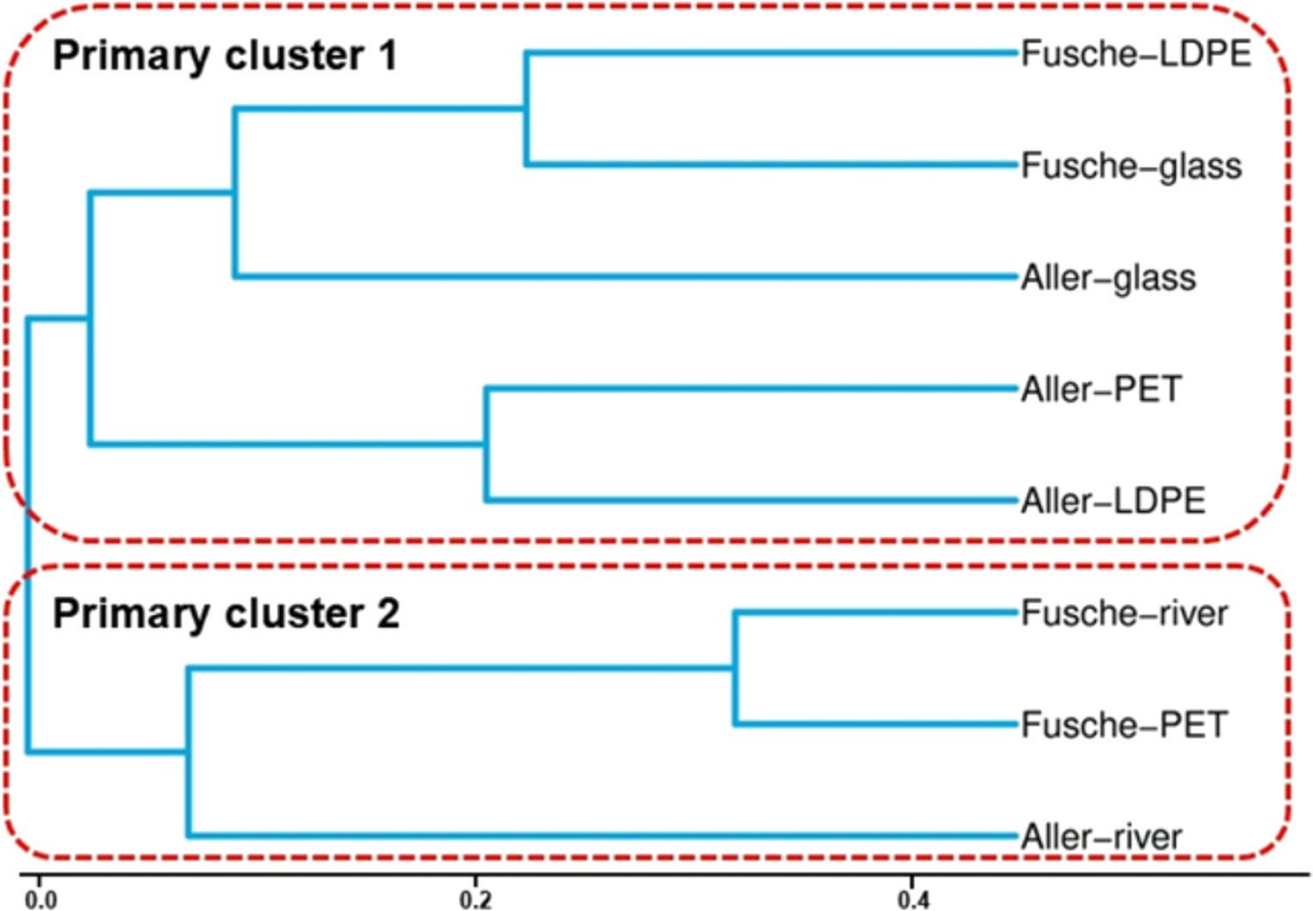
UPGMA (Unweighted pair group method with arithmetic mean) dendrogram illustrating the hierarchical clustering of microbial communities across different samples based on Bray-Curtis dissimilarity. Each branch represents an individual sample, and the clustering reflects the similarity in community composition between samples, highlighting site- and plastic-specific grouping patterns

The UPGMA (Unweighted pair group method with arithmetic mean) clustering method was used to cluster the samples based on the Bray-Curtis distance matrices. The hierarchical clustering analysis of microbial community composition profile across different surfaces (plastic and glass) and river water samples revealed distinct patterns shaped by site-specific microbial composition and surface types (Fig. 3). The dendrogram clearly delineates two primary clusters corresponding to the two sampling sites, one which contains Fusche river, Fusche- PET, and Aller river and the other one consisting the remaining treatments.

Within each sample type, substrate type further influenced microbial community composition. Microbial communities associated with LDPE and glass incubated in the Fusche river water clustered together, indicating a similar microbial colonization pattern on these inert substrates. In contrast, PET and Fusche river water formed a separate sub-cluster, reflecting differences in biofilm development or substrate activity.

Microbial communities on glass, PET, and LDPE incubated in Aller river water are grouped, showing a relatively high degree of similarity. This suggests that the riverine microbial seed pool may be more influential in shaping biofilm communities on these substrates, overriding substrate-specific effects to some extent. However, the Aller River sample (the free-living microbial community) branched off distinctly from all other samples. This indicates that the riverine communities differ significantly from biofilm-associated communities, emphasizing the selective nature of substrate colonization and the establishment of niche- specific microbial assemblages (Dang and Lovell 2016).

The divergence of the Aller River also underscores the ecological differences between surface-attached and free-floating niche microbial communities, possibly driven by factors such as nutrient availability of the medium and surface chemistry of the substrate (Battin et al. 2016). The uniqueness of this cluster may also reflect the presence of transient or opportunistic microbes in river water that are not efficient colonizers of solid surfaces (Amaral-Zettler et al. 2020). Overall, the dendrogram suggests a combined influence of niche microbial assemblages and substrate on the microbial community structure. These findings support the idea that while substrate type governs the initial attachment and community selection, it is the water chemistry and microbial seed pool that ultimately modulate the community composition over time. Such insights are crucial in understanding microbial succession, substrate-specific colonization dynamics, and the development of plastisphere communities in freshwater ecosystems (Oberbeckmann et al. 2015).

#### Taxonomic composition of bacterial communities across samples

**Fig. 4:**
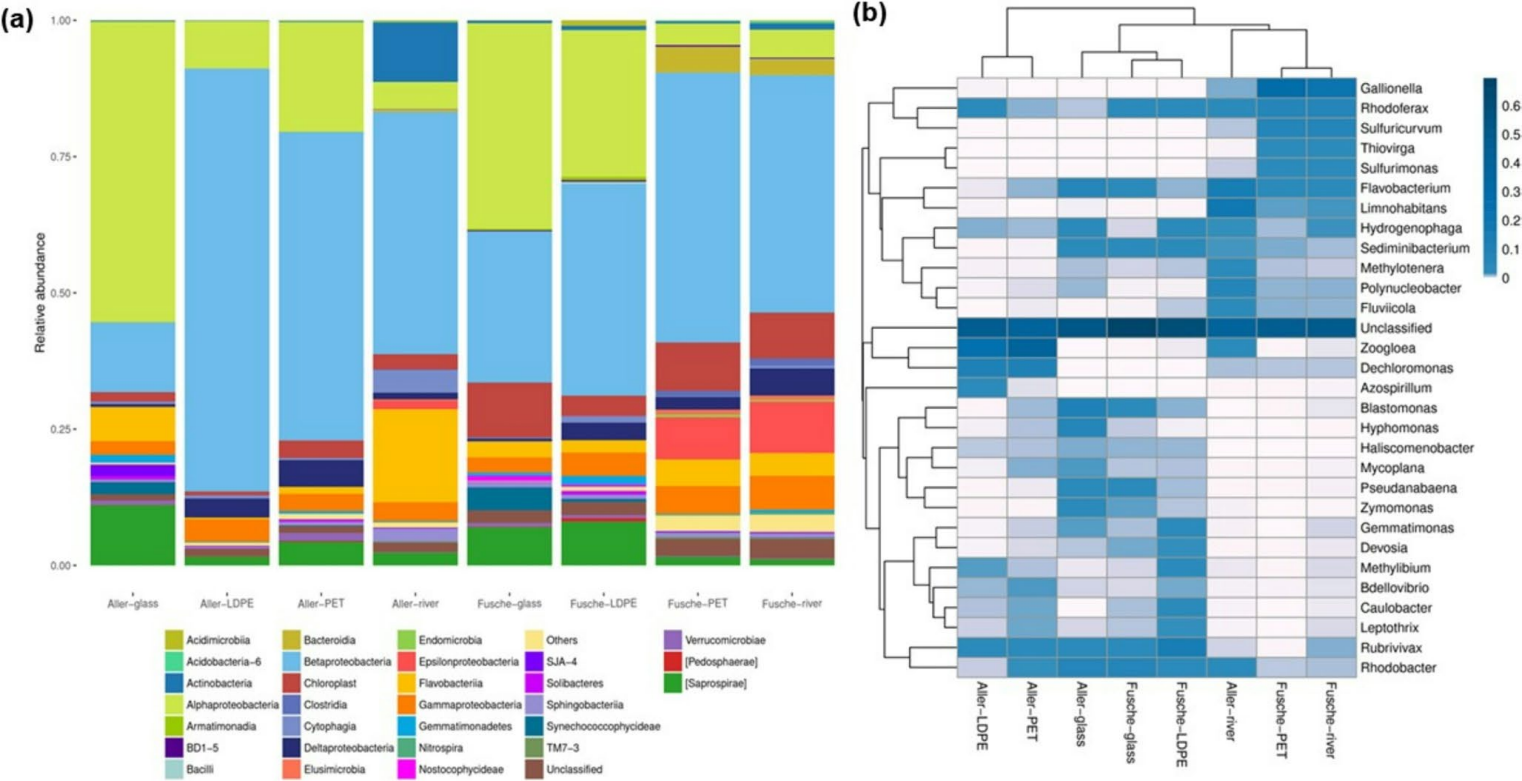
**a** Bar plot showing the distribution of the top 30 most abundant classes based on relative abundance across the samples; **b** Heatmap depicting the relative abundance of the top 30 bacterial genera, clustered based on similarity. Colour gradient represents abundance variations, illustrating compositional similarities and differences among samples at the genus level

Figure S3 presents a detailed taxonomic composition of the different samples. Figure 4 shows the distribution of the top 30 most abundant class- and genus-level classifications in each sample. Bar charts plot the distribution of the top 30 most abundant classifications in each sample or group at the class level with respect to their relative abundance (ra) (Fig. 4a). The top 30 genus distributions for each sample were clustered and plotted in a heatmap (Fig. 4b). The heatmap (Fig. 4b) uses a colour scheme (Dark blue to white) to visualize the similarities and differences of each species.

Microbial communities associated with river water and substrate-specific biofilms showed distinct compositional patterns shaped by both site- specific environmental factors and substrate characteristics (Fig. S3). Proteobacteria (0.6–0.9 ra), Bacteroidetes (0.2–0.3 ra) were the most dominant phyla across all samples from the Aller and Fusche rivers (Fig. S4), consistent with previous findings in freshwater systems where these groups are often linked to nutrient cycling and biofilm formation (Zeglin 2015; Zhai et al. 2023). Interestingly, Actinobacteria (~ 0.2 ra) were more abundant in the Aller river, whereas Cyanobacteria (~ 0.1 ra) showed higher prevalence in Fusche samples, suggesting underlying ecological or physicochemical differences between the two rivers (Fig. S4). Despite overall similarities, the Cyanobacteria fraction in Fusche was consistently higher across both river water and plastic surface, pointing toward a potential influence of light exposure and nutrient availability in shaping local community structures.

Material-associated communities exhibited clear differentiation, particularly between biofilms on synthetic polymers (PET and LDPE) and those on glass or in river water (Fig. 4 and S2). Betaproteobacteria (varies between 0.3 and 0.8 ra), especially from the Burkholderiales and Rhodocyclales orders, were highly abundant on PET and LDPE surfaces. It reflects their own affinity for artificial substrates and capacity for robust biofilm development (Oberbeckmann et al. 2015; Kirstein et al. 2018). The Aller PET and LDPE samples, in particular, stood out as distinct from all others due to a strong enrichment of *Rhodoferax*, a genus known for its metabolic flexibility and association with plastic surfaces (Suyama et al. 1998). In contrast, glass substrates hosted higher levels of Gemmatimonadales, which have been previously reported in freshwater benthic biofilms but are less commonly found on plastics (Flemming and Wingender 2010). Interestingly, the community associated with PET incubated in Fusche river water closely resembled the Fusche river water community, indicating that environmental factors may override substrate influence in some contexts, whereas the community associated with LDPE incubated in Fusche river water diverged significantly, emphasizing substrate- specific microbial selection (Reis and Aldridge 2024). The Comamonadaceae family was ubiquitous across all samples, underscoring its adaptability and likely role as a core member of freshwater microbial assemblages. These findings echo patterns observed in both marine and freshwater studies (Pinto et al. 2019; Zhang et al. 2022; Delacuvellerie et al. 2022; Li et al. 2022; Zhu et al. 2023), which report similar dominance of Proteobacteria and Bacteroidetes on plastic surfaces, but also highlight the distinct community assembly dynamics in rivers due to fluctuating hydrology, land-use inputs, and microbial dispersal.

A finer taxonomic resolution, unclassified genera dominated across all samples (Fig. 4), revealing both the high diversity and the limitations of current databases in resolving freshwater plastisphere communities. Among the classified taxa, *Flavobacterium* emerged as a consistently dominant genus in both river systems, known for its role in degrading high-molecular-weight organic matter (Szabó et al. 2021). *Sediminibacterium* was found in all the samples except in the Aller river community and PET samples from the Aller river (Fig. 4). Both these genera belong to Bacteroidetes, which is a common Phylum associated with early biofilm development on plastic surfaces. Overall, the differences observed between rivers and substrates in this study point to the complex interplay between local environmental conditions, substrate type, and ecological functional potential in shaping microbial community structure on natural and artificial surfaces in freshwater systems (Ogonowski et al. 2018).

Recent works also highlight the functional and structural implications of plastic colonization beyond taxonomy. A long-term freshwater mesocosm study showed that even after several months, MP biofilm communities remain highly dynamic and increasingly dissimilar across polymer types, with notable enrichment of taxa like *Devosia* on polyolefins (Tagg et al. 2022). In another study, Wallbank et al., reported the presence of *Flavobacterium* sp. and *Rhodoferax* sp. as potential plastic degraders within the microbial network in the plastisphere and on glass, respectively, in the final polishing pond of a wastewater treatment plant (Wallbank et al. 2025).

#### Diversity of the bacterial communities

The diversity of bacterial communities was assessed using rarefaction curves and an abundance rank plot, revealing variations in species richness and dominance across samples.

**Fig. 5:**
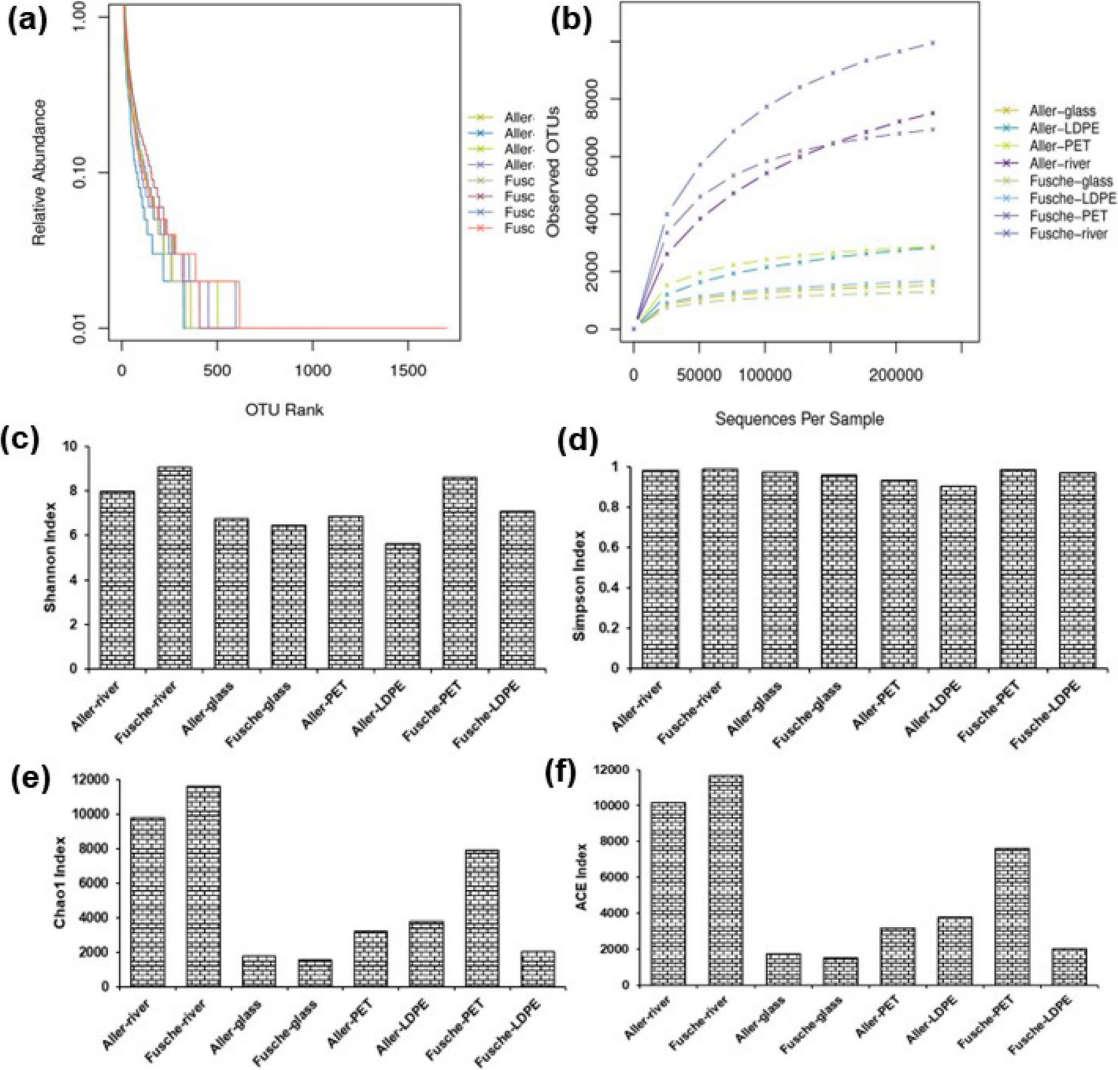
**a** Rank abundance curves of OTUs for each sample. The X-axis shows OTU rank in descending order of abundance, and the Y-axis shows the relative abundance (of total sequences) for each OUT; **b** Rarefaction curves showing OUT richness versus number of sequences per sample. Plateauing curves indicate sufficient sequencing depth for community analysis. (**c–f**) Bar plots representing alpha diversity metrics, including **c** Shannon, **d** Simpson, **e** Chao1, and **f** ACE indices across different samples, illustrating variations in microbial diversity and richness

The abundance rank curve (Fig. 5a) reveals that microbial communities across all the samples are dominated by a few highly abundant Operational taxonomic units (OTUs), followed by a long tail of low abundance taxa, indicating typical ecological structuring with high richness but uneven distribution. It is a hallmark of microbial ecosystems where early colonizers dominate, particularly during initial biofilm formation. The pattern observed is consistent with reports from the other aquatic biofilm studies, where fast-growing or material-specific bacteria rapidly outcompete others during early succession stages (Di Pippo et al. 2022). The overlapping curves across different substrates and river water samples suggest broadly similar community abundance patterns, with substrates having limited influence on dominance structure. Notably, the Fusche river sample shows a slightly extended curve with a gentler slope, reflecting higher richness and a more even community, likely due to the absence of surface-specific selection pressure present in biofilms. This suggests that the natural river water sample supports a broader and more balanced microbial assemblage compared to synthetic materials.

Similarly, the rarefaction analysis (Fig. 5b) revealed that microbial richness was highest in river water samples, especially from Fusche, highlighting the natural river environment as a niche for diverse microbial communities. PET and LDPE materials supported moderate richness, suggesting these plastics offer niche surfaces for microbial colonization, while glass showed the lowest diversity due to inertness, which validates that inert substrates have biofilm complexity due to limited physical heterogeneity (Rummel et al. 2017; Sooriyakumar et al. 2022). The site-specific effects were evident, with Fusche generally hosting more diverse communities than Aller, likely influenced by environmental factors such as nutrient levels or pollution. Most curves plateaued, confirming sufficient sequencing depth, albeit the Fusche river samples still showed an upward trend, indicating rare taxa were still being detected. This pattern underlines rivers as microbial reservoirs, consistent with findings reported by Li et al. (2022), who reported elevated microbial diversity in surface water bodies with plastics (Li et al. 2022). Colin O’Donnell, in their thesis, also reported similar richness in coastal marine plastisphere studies of beached nurdles (Donnell 2023).

Alpha and beta diversity analyses further highlighted the differences in within-sample complexity and between-sample community structure, respectively, emphasizing the impact of material type and environmental factors.

##### Alpha diversity

Alpha diversity indices reveal important insights into how microbial richness and evenness vary between river water medium and substrate-associated biofilms. The Shannon index (Fig. 5c), which reflects both species richness and evenness, was highest in the Fusche river sample, followed by the Aller river and the Fusche- PET, indicating a more diverse and balanced microbial community in river water medium compared to biofilms present on the substrates. Notably, the lowest Shannon values were observed in Aller- LDPE and Fusche- glass, suggesting a reduced microbial complexity on artificial surfaces, particularly on glass and LDPE. These patterns align with findings from recent studies, such as Erni-Cassola et al. (2020) which showed that biofilm formation on inert substrates like glass can result in limited diversity due to weaker surface attachment and slower colonization rates compared to naturally suspended microbial communities (Erni-Cassola et al. 2020).

The Simpson index (Fig. 5d), which emphasizes community evenness, remained high and relatively consistent across all samples, implying that despite differences in richness, the distribution of taxa within each sample was fairly even. The minor fluctuations observed suggest that substrate type or river origin had a lesser impact on evenness than on richness. This agrees with the observations by Zhao et al. (2024), who reported that while substrate-associated microbial communities show clear differences in richness and taxonomic composition, the relative distribution of dominant taxa often remains even due to ecological niche saturation within confined biofilm environments (Zhao et al. 2024).

Both Chao1 and ACE indices (Fig. 5e and f), which estimate species richness by accounting for rare or less abundant taxa, further highlighted that natural river water samples, particularly from the Fusche, harboured the highest microbial richness, followed closely by the Aller river. In contrast, richness was significantly lower in substrate-associated communities, with glass and LDPE supporting the least diverse microbiota. Among plastic substrates, Fusche- PET retained notably higher richness, suggesting that PET may support more heterogeneous microhabitats than LDPE, likely due to differences in surface texture, hydrophobicity, or nutrient adsorption potential. Similar findings were reported by Song et al. (2022), where PET consistently supported a more diverse microbial community than polyethylene-based substrates in freshwater environments (Song et al. 2022).

Overall, these diversity matrices highlight that natural environments promote richer and more complex microbial assemblages, while synthetic substrates, especially glass and LDPE, tend to support less diverse communities. PET, however, emerges as an intermediate, capable of retaining higher richness, possibly due to its compatibility with certain biofilm-forming taxa. These patterns underline the importance of substrate material and local environmental context in shaping microbial colonization and community development in aquatic plastisphere studies (Oberbeckmann et al. 2016; Zhao et al. 2024) .

##### Beta diversity

Beta diversity reflects the diversity and the degree of differences among samples. The distance between the samples was calculated using the evolution and abundance information between the sample sequences to reflect whether there is a significant difference in microbial community among the samples. This was achieved by UniFrac analysis.

**Fig. 6:**
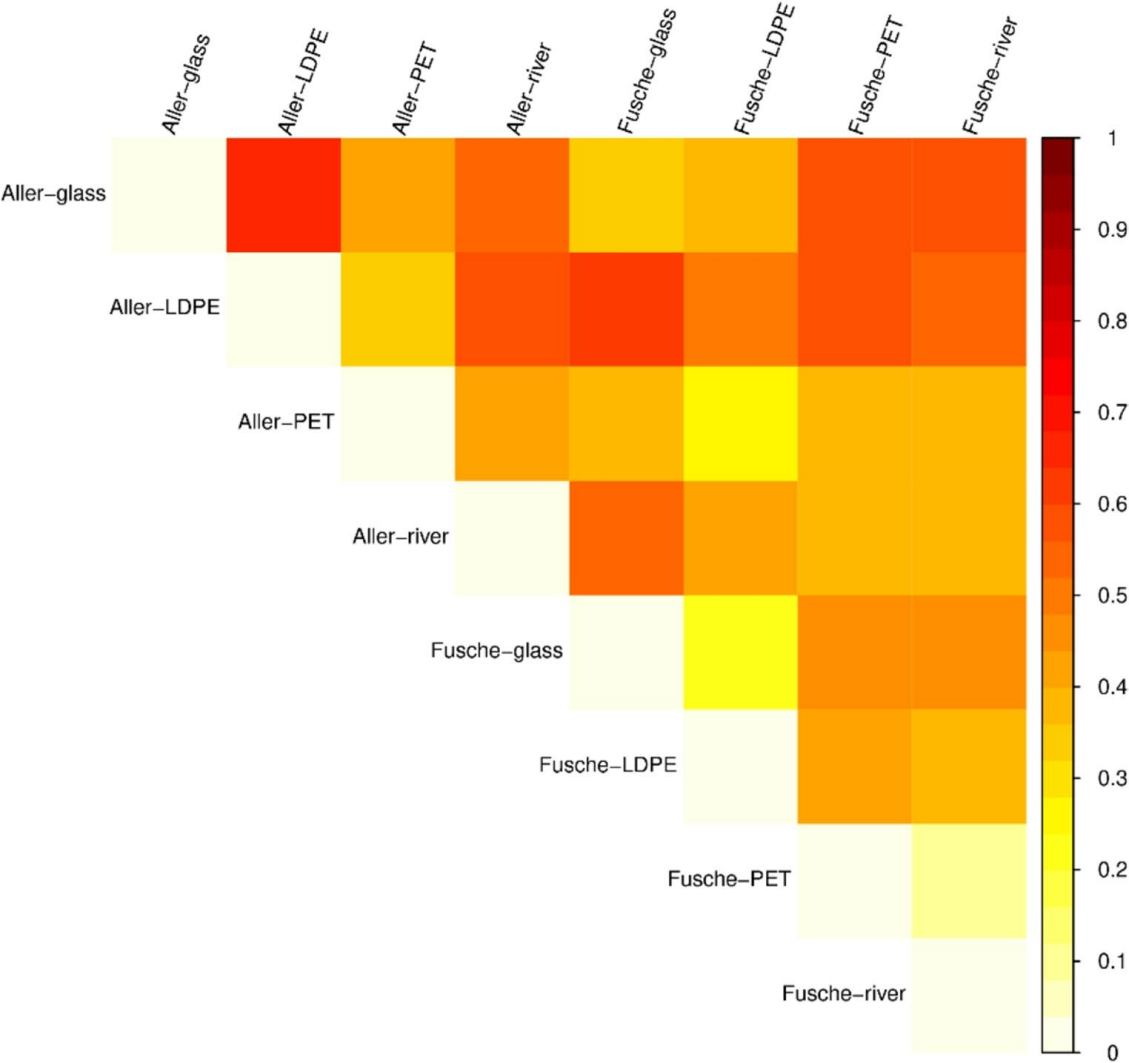
Heatmap of (un)weighted UniFrac distance matrix representing beta diversity among samples. Colour intensity indicates the degree of dissimilarity; a lighter colour reflects lower distances and more similar microbial communities between samples

The Bray-Curtis dissimilarity heatmap (Fig. 6) provides insight into the beta diversity and compositional shifts across different samples. Substrate-specific clustering was evident, as samples from the same material type tended to group more closely. Notably, Aller- PET and Aller- LDPE exhibited high similarity (darker orange tones), suggesting a shared microbial assemblage likely influenced by similar polymer characteristics and environmental exposure. Fusche-PET and Fusche-LDPE also displayed moderate similarity, reinforcing the notion that substrate type governs community composition. In contrast, Aller- glass and water sample from Fusche appeared most dissimilar from the rest (yellow to white), indicating distinct microbial communities, possibly due to the inert nature of glass limiting biofilm development, and river water representing a planktonic, free-living microbial community with greater variability. These patterns emphasize that both substrate material and site location shape microbial community structure, with synthetic polymers supporting more convergent microbial profiles than either glass or natural river water environment (Oberbeckmann et al. 2016).

**Fig. 7:**
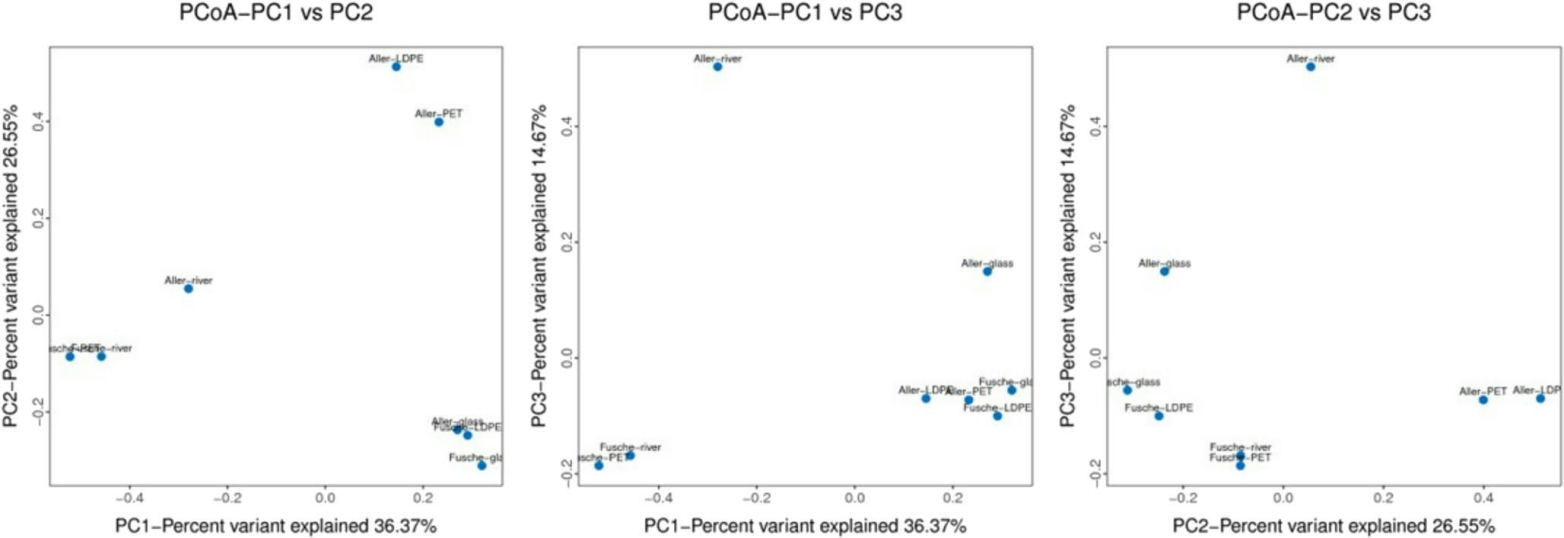
Principal Co-ordination Analysis (PCoA) plot based on the first (PC1) and second (PC2) principal coordinates. The X- and Y- axes represent the proportion of total variance explained by each coordinate. Shorter distances between sample points indicate higher similarity in microbial community composition, with clustered samples reflecting similar taxonomic profiles

The Principal Coordinates Analysis (PCoA) plots (Fig. 7) based on Bray-Curtis dissimilarity revealed clear spatial separation in microbial community composition driven predominantly by the indigenous microbial community rather than the material type. The first three axes (PCoA1, PCoA2, PCoA3) account for a cumulative 77.6% of the variation, indicating a robust representation of community dissimilarity. Fusche-PET and Fusche river samples consistently cluster together across all the plots, suggesting that environmental factors in the Fusche river, such as water quality or pollutant exposure, strongly influence microbial assemblages, even across different substrates. In contrast, Aller- PET and Aller- LDPE cluster distinctly on the positive side of PC1 and PC2, reinforcing site-specific divergence from Fusche counterparts. The Aller river sample stands out as an outlier along PC3, indicating a unique microbial characteristics not captured by PC1 or PC2, possibly reflecting distinct ecological pressures or microbial processes in the river water. Notably, glass samples do not form a cohesive group, with Aller-glass and Fusche-glass occupying different quadrants, further emphasizing that the site exerts a stronger influence than substrate type on microbial community structure. These trends suggest that riverine microbial communities respond more strongly to local physicochemical conditions than to differences in substrate surfaces alone, aligning with findings from previous studies on freshwater plastisphere dynamics (Zhu et al. 2023; Zhao et al. 2024).

### Mechanisms of microbial attachment and biofilm formation on plastic materials

The process of microbial colonization on plastic and other surfaces begins with the formation of a conditioning film. This film is a layer of organic and inorganic molecules that rapidly adheres to a clean surface when it comes into contact with water or a biological medium. This film alters the surface morphology, making it more favourable for microbial attachment by providing nutrients, changing hydrophobicity, and charge interactions (Rummel et al. 2017; Shineh et al. 2023; Zhang et al. 2024). Initial adhesion occurs when microbial cells encounter the conditioned surface and attach through weak physical forces such as van der Waals interaction and electrostatic interactions (Ploux et al. 2010). Microbial surface structures like flagella, pili, and fimbriae strengthen attachment by mediating specific interactions between cells and the material, particularly on hydrophobic polymers like PE (Zhang et al. 2023).

Once a critical number of cells have accumulated, quorum sensing (QS) becomes pivotal. QS is a chemical communication system in which cells produce and detect small signal molecules known as autoinducers (Erkihun et al. 2024). When cell density increases sufficiently, autoinducer concentrations rise and trigger coordinated changes in gene expression that regulate extracellular polymeric substance (EPS) synthesis and the biofilm-associated functions. This signalling shifts the community from reversible attachment to irreversible adhesion and promotes the transition to structured microcolonies. As the biofilm matures, cells are embedded within this matrix and develop channels that allow the distribution of water, nutrients, and waste. At later stages, cells detach, contributing to dissemination (Markowska et al. 2024; Ugwu et al. 2025). Altogether, surface conditioning, physicochemical properties of the material, microbial adhesion, QS regulation, and EPS production drive the formation of biofilms on plastics and other surfaces (Flemming et al. 2025).

### Do biofilm-forming bacterial assemblages reflect surface-specific ecological pressures and functional niches?

The substrate-specific microbial communities observed in this study reflect adaptive responses to the physicochemical properties of PET, LDPE, glass, and the river water, with implications for functional ecology and biogeochemical cycling (Seeley et al. 2020). While Proteobacteria and Bacteroidetes dominated most samples, consistent with their known roles in nutrient cycling and organic matter degradation, specific differences emerged between the substrates (Dey et al. 2022; Feng et al. 2023). Proteobacteria is widely recognized as a key contributor to biogeochemical nitrogen cycling due to its genetic and metabolic versatility, and its dominance on plastic-associated biofilms suggests that plastics act as favourable ecological niches for nitrogen-transforming microorganisms. The enrichment of nitrogen-cycling taxa on plastics indicates that these materials function not only merely as substrates, but as microbial hotspots for localized nitrogen transformations like nitrogen fixation, dissimilatory nitrate reduction to ammonia (DNRA), and ammonification (Feng et al. 2023). They often dominate plastisphere communities with genera capable of degrading complex organic matter. They are enriched on plastic surfaces because they can utilize hydrocarbons and other plastic-derived compounds as carbon sources (Cholewińska et al. 2025). Synthetic polymers, PET and LDPE, consistently supported higher abundances of Betaproteobacteria, particularly those in the Burkholderiales and Rhodocyclales orders, known for their biofilm-forming abilities and metabolic flexibility on synthetic surfaces (Miao et al. 2021). Genera such as *Rhodoferax*, *Rubrivivax*, and *Hydrogenophaga* were notably enriched on these materials, indicating potential for nitrogen cycling, hydrogen oxidation, and even degradation of plastic- associated compounds (Willems et al. 1989; Suyama et al. 1998; Curtis 2016).

Glass substrates, on the other hand, were dominated by Rhodobacteraceae, Rhodocyclaceae, and Gemmatimonadaceae (Fig. S5), which are very common in natural benthic or sediment attached communities, possibly due to their adaptation to inert and hydrophilic surfaces (Bocci et al. 2024). These Alpha proteobacteria are adapted to photoheterotrophic metabolism and early biofilm development via EPS production and oxidative stress tolerance (Dey et al. 2022). Comamonadaceae was widespread across all materials and water samples (Fig. S5), indicating its ecological versatility and possible function as a core biofilm constituent in freshwater systems (Beltrán de Heredia et al. 2025). Additionally, *Stramenopiles* (heterokonts, notably present in PET samples from both river water) suggest a co-colonization by eukaryotic microbes, which may influence bacterial biofilm structure and succession. It enhances structural complexities and nutrient exchange in developing plastisphere communities (Jirsová and Wideman 2024). Microbes from the family Flavobacteraceae (Fig. S5), particularly the genus *Flavobacterium* was ubiquitous across both substrate and river water samples. These taxa are proficient in breaking down complex organic matter via enzymatic activity (such as proteases, glucanases), contributing to biofilm structural integrity and carbon cycling (Kang et al. 2013). Members of the Oxalobacteraceae family enriched on Aller LDPE and Fusche PET are reportedly helpful in organic acid metabolism and surface conditioning on inert or polymeric substances (Baldani et al. 2014) by expressing depolymerases, esterases, and related enzymes that target polymer structures, contributing to partial plastic deterioration (Lv et al. 2024). They often participate in nutrient cycling under oligotrophic conditions. Meanwhile, *Sediminibacterium*, dominant across all samples except polymers incubated in Aller river water, is associated with particulate organic matter degradation and community stabilization through EPS production and biofilms (Qu and Yuan 2008). It also indicates that plastic surfaces may alter microbial selection or suppress certain freshwater taxa through competitive exclusion or lack of suitable niches. Additionally, the presence of *Rhodobacter* (In all PET and LDPE substrates) indicates that plastisphere communities may harbour bacteria capable of hydrogen metabolism, photoheterotrophy, or even xenobiotic degradation (Sun et al. 2023).

### Significance and prospects

The study presents a detailed exploration of microbial community structures colonizing various substrates like PET, LDPE, and glass in two distinct riverine samples (Aller and Fusche), revealing how substrate characteristics and niche microbial seed pool influence bacterial diversity and potential ecological functions. The integration of taxonomic profiles and diversity indices provides a broader view of the early colonization of biofilm on different materials. The study advances our understanding of how substrate type and site-specific water quality shape biofilm-forming microbial communities in freshwater microcosms. The observed enrichment of functionally diverse bacteria ranging from organic matter degraders like *Flavobacterium* and *Sediminibacterium* to metabolically flexible taxa like *Rhodoferax*, *Rubrivivax*, and *Hydrogenophaga* highlights the potential for plastics to serve not only as passive surfaces but also ecological niches that influence microbial succession, nutrient cycling, and pollutant transformation. The microbial signatures observed on these synthetic substrates suggest selective colonization by the microbial community on the synthetic interface that favours taxa with specialized survival and degradation capabilities (Miao et al. 2021). From a broader ecological and environmental standpoint, these findings emphasize the role of plastics as vectors for microbial dispersal and hotspots of biogeochemical activity, especially in lotic ecosystems (Bižić-Ionescu et al. 2018). Understanding the taxonomic and functional profiles of plastisphere communities may aid in predicting ecosystem responses to plastic pollution and identifying microbial indicators of anthropogenic disturbances.

Looking ahead the future research could explore the meta-transcriptomics or meta-proteomics to uncover active metabolic pathways on different substrates (Panicker 2024), as the functional niches are discussed in this study only based on the literature. As the biofilm communities harbour microbes from alpha, beta, delta, and gamma proteobacteria abundantly, bioprospecting for plastic-degrading enzymes (PDEs) can be done. The study provides a static perspective in a river water microcosm, but biofilm development is dynamic and influenced by succession. So, in situ exploration of plastisphere dynamics on daily-use plastics can provide a clearer picture regarding the microbial communities. Plastisphere dynamics can be predicted under varying hydrological and pollutant levels by modelling community assembly. These directions may ultimately support strategies for plastic bioremediation, microbial risk assessment, and eco-design of biodegradable polymers tailored to mitigate long-term environmental impacts.

## Conclusion

The study provides a comprehensive overview of microbial communities in natural river water and substrate-attached biofilms in two contrasting freshwater environments. The findings highlight the critical roles of substrate type and site-specific environmental conditions in shaping bacterial community structure and diversity. Plastics such as PET and LDPE supported moderate microbial richness and harboured functionally distinct bacterial taxa. This suggests that these synthetic surfaces can act as selective ecological niches that potentially influence biodegradation and nutrient cycling. Due to its inert nature, glass supported the least diverse communities. River water, particularly from the Fusche site, emerged as a biodiversity hotspot. Bacterial families such as Flavobacteraceae, Comamonadaceae, and Rhodobacteraceae played prominent roles, reflecting metabolic versatility and potential ecological functions ranging from organic matter degradation to biofilm stability. This work contributes to the growing body of evidence on the freshwater plastisphere and underscores the necessity of further functional and temporal studies to better comprehend the ecological ramifications of microbial colonization on plastic surfaces within dynamic aquatic ecosystems.

## Supplementary Information

Supplementary Material 1.

## Data Availability

The data can be made available upon reasonable request to the corresponding author.
