# Supplementary material for: Early microbial colonization study of daily-use plastics exposed to river water
Source: World J Microbiol Biotechnol. 2026 Apr 18;42(5):218. doi: 10.1007/s11274-026-04907-z (PMC13090205; doi:10.1007/s11274-026-04907-z)
Supplement: Supplementary file 1 — Supplementary Material 1. [file 11274_2026_4907_MOESM1_ESM.docx]

**Supplementary Information**

**Early Microbial Colonization Study of Daily-use Plastics Exposed to River Water**

Dinesh Parida^a, b, 1^, Swagata Lakshmi Dhali^a, b^, Kiran Bala^c, 2^, Regina Nogueira^b, 3*^

^a^Mehta Family School of Biosciences and Biomedical Engineering, Indian Institute of Technology Indore, Simrol, Madhya Pradesh, India, 453552.

^b^Institute of Sanitary Engineering and Waste Management, Leibniz University Hannover,

Welfengarten 1, Hannover, Germany, 30167.

^c^Mehta Family School of Sustainability, Indian Institute of Technology Indore, Simrol, Madhya Pradesh, India, 453552.

E-Mail Address: [phd2101271003@iiti.ac.in](mailto:phd2101271003@iiti.ac.in), [swagatadhali1011@gmail.com](mailto:swagatadhali1011@gmail.com), [kiranb@iiti.ac.in](mailto:kiranb@iiti.ac.in)

*Corresponding Author E-Mail Address: nogueira@isah.uni-hannover.de

^1^0000-0003-2299-1776

^2^0000-0002-0939-5809

^3^0000-0003-2759-5035


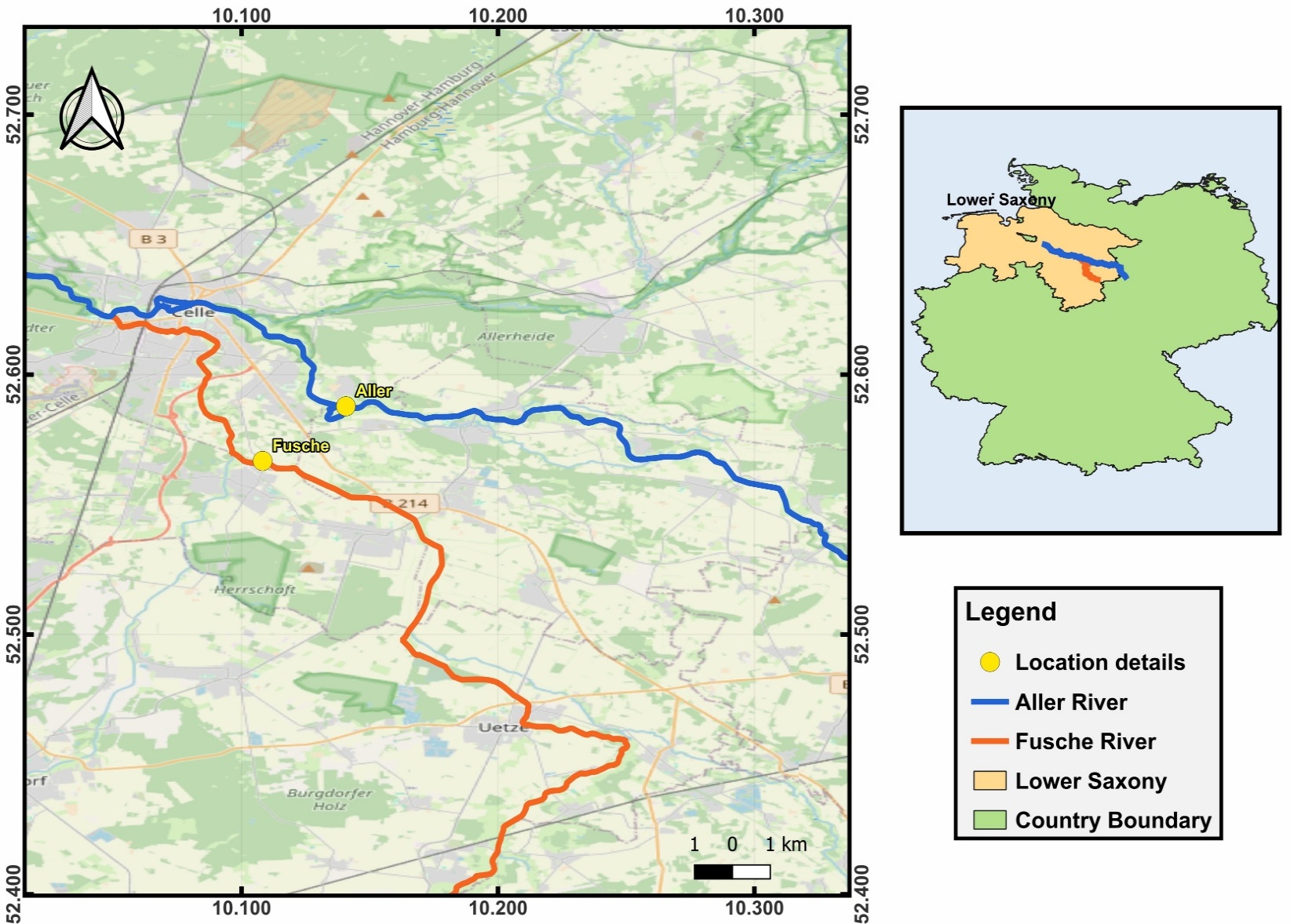


**Fig. S1** An Overview of the locations where the samples were collected


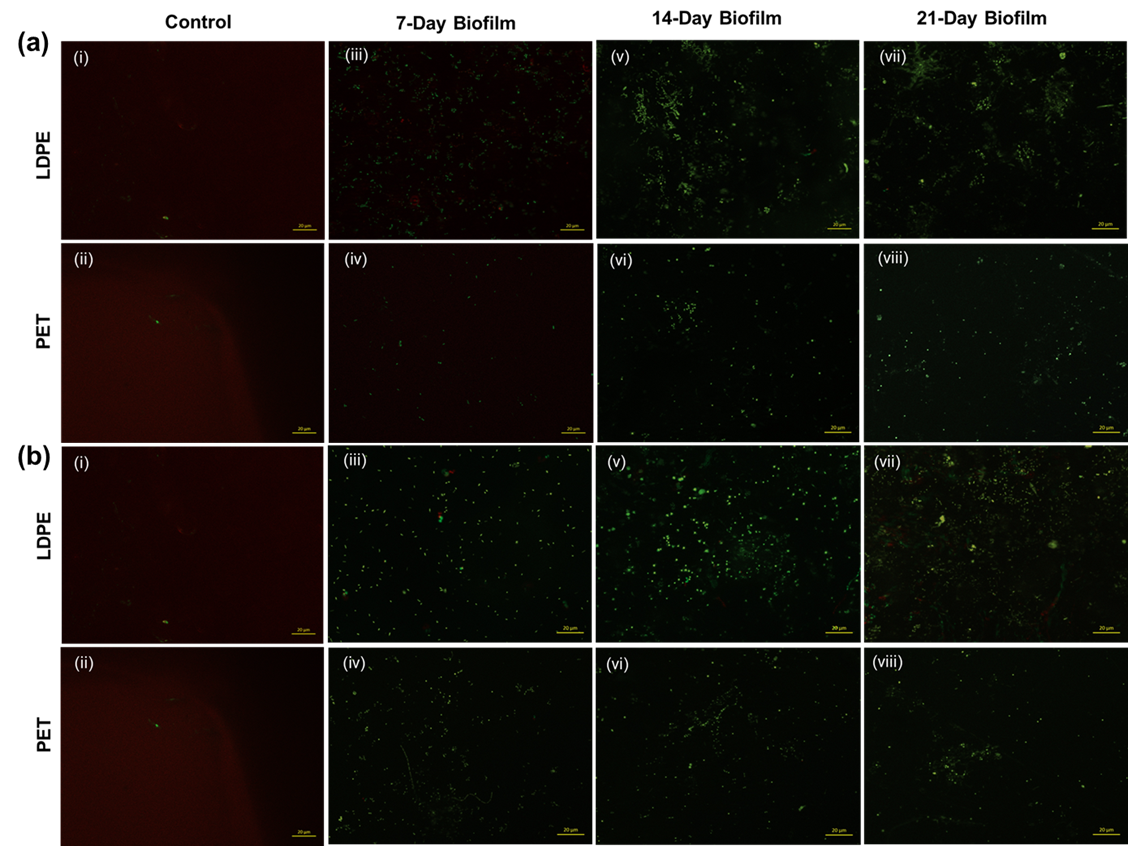


**Fig. S2** Fluorescence micrographs showing biofilm formation on LDPE and PET microplastic surfaces incubated in river water. Progressive increase in fluorescence over time indicates enhanced microbial colonization on both polymer types in (a) Aller River water and (b) Fusche River Water; with visible differences influenced by polymer surface properties and river-specific microbial communities


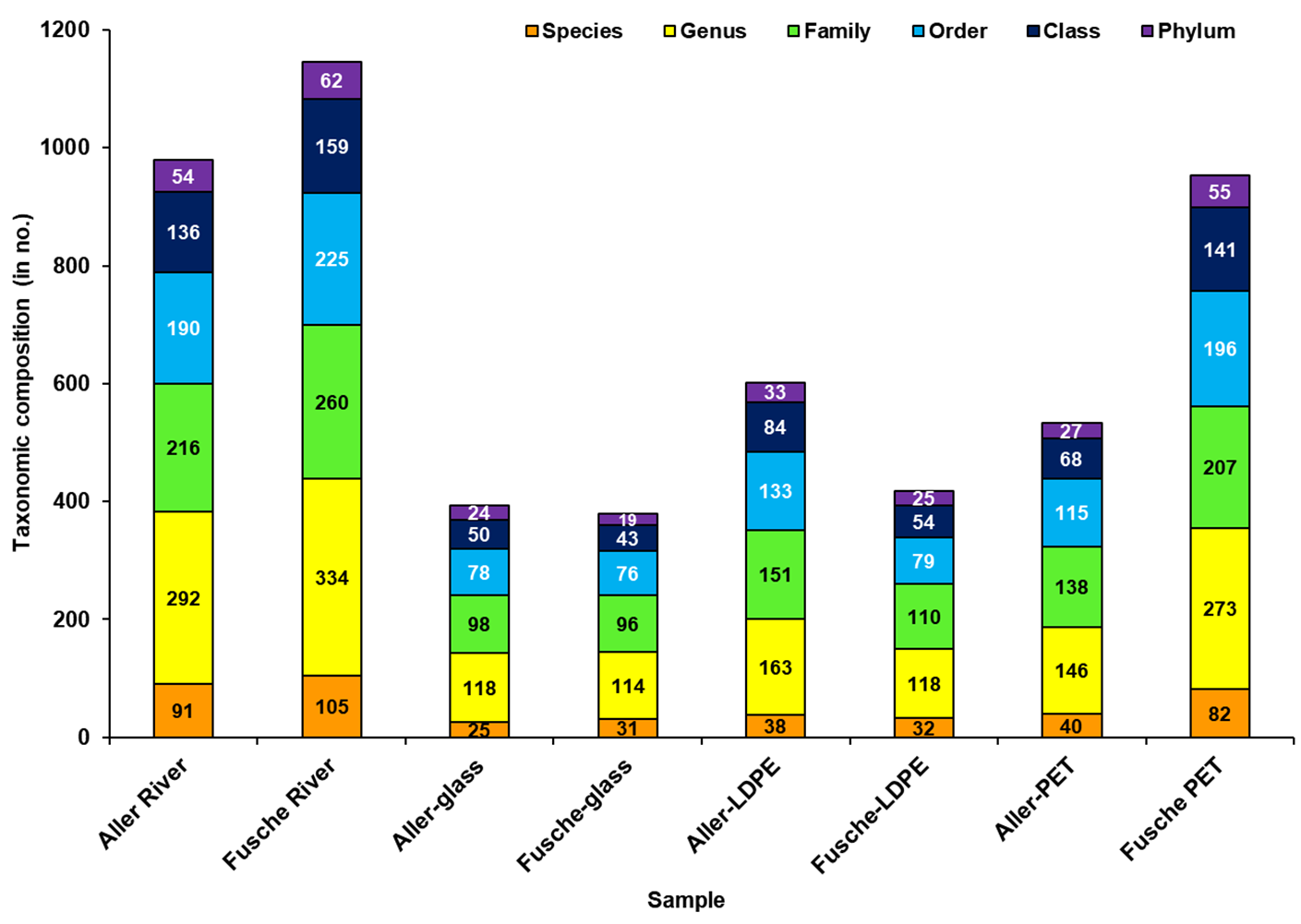


**Fig. S3** Histogram illustrating the overall taxonomic composition across different hierarchical levels for each individual sample


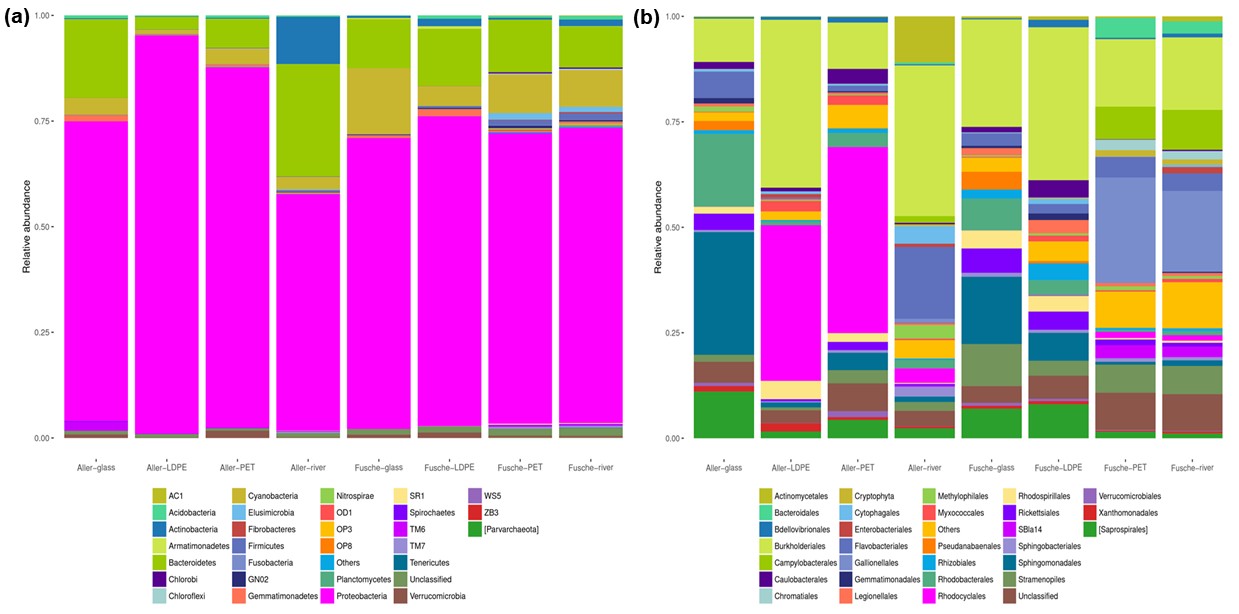


**Fig. S4** Bar plots showing the relative abundance patterns of the top bacterial (a) phyla and (b) orders across all samples


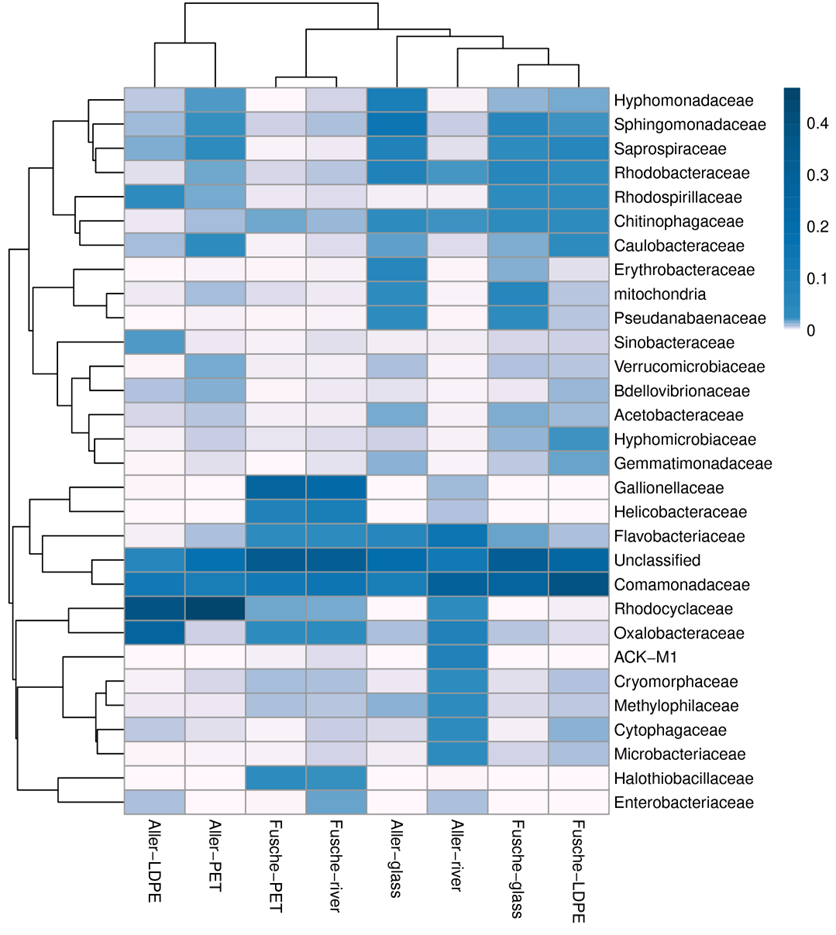


**Fig. S5** Heatmap depicting the relative abundance of the top 30 bacterial families, clustered based on similarity. Colour gradient represents abundance variations, illustrating compositional similarities and differences among samples at the genus level
